# Stratified/risk-based screening for colorectal cancer in the UK: an overview

**DOI:** 10.1080/1758194X.2025.2501851

**Published:** 2025-05-20

**Authors:** Susanne Maxwell, David Weller, Becky Dennison, Lily Taylor, Hannah Miles, Joanne Cairns, Christian Von Wagner, Jazzine Smith, Juliet Usher-Smith

**Affiliations:** a University of Edinburgh, Usher Institute, Edinburgh, UK; b Department of Public Health and Primary Care, University of Cambridge, Cambridge, UK; c Hull York Medical School, Academy of Primary Care, Hull, UK; d University College London Institute of Epidemiology & Health, London, UK

**Keywords:** Colorectal cancer, stratified screening, personal bowel cancer risk, genetic testing, risk-based colorectal screening

## Abstract

While colorectal cancer screening is well-established in the UK, at present, it uses a ‘one-size fits-all’ approach – that is, individual risk is not considered when determining screening regimens (except for some specific conditions such as Lynch syndrome). Stratified screening offers the prospect of directing more intensive screening toward those at higher risk – with a corresponding reduction of screening intensity among lower-risk individuals. Two key opportunities for stratifying colorectal cancer screening are (1) making better use of an individual’s quantitative fecal hemoglobin result rather than the current approach of deeming tests to be positive over an arbitrary threshold and (2) gathering information on lifestyle, family history, genetics and other factors to establish risk of colorectal cancer – and using this information to tailor screening regimens. While there is encouraging evidence from modeling studies demonstrating reduced colonoscopy requirements and increased positive prediction of colorectal cancer when incorporating risk assessments within screening, we need ‘real world’ evidence on stratified screening to establish whether it is effective, improves screening outcomes and is acceptable to the public. We also need to know the impact these changes would have on existing screening programs, and how programs might adapt their organizational and IT processes.

## Plain Language Summary

Bowel cancer is the fourth most common cancer in the UK. Bowel screening aims to find cancer early or find changes in the bowel (polyps) before they develop into cancer. The bowel screening test is called fecal immunochemical test (FIT) and is completed at home and sent away to detect tiny traces of blood in the stool. The level of these traces of blood is called the fecal hemoglobin (f-HB) level. If the f-HB level is above a set amount patients will be offered further testing which may include a colonoscopy.

Not all people have the same risk of bowel cancer – some people have a higher risk, some lower. Despite these differences in risk, at present, everyone gets offered the same screening. There could be options to improve screening based on a person’s unique risk. This could be through increasing screening in higher risk people or reducing screening for people who are lower risk. Deciding an individual’s risk could be done in two ways (1) using the f-HB level rather than a ‘positive or ‘negative’ result. (2) Using lifestyle or personal factors such as their age or sex or their genetic make-up.

This article reviews the evidence for changing the screening process based on an individual’s risk and what further information is needed before this can be put in place.

## 1. Introduction

Colorectal (bowel) cancer is diagnosed in over 42,000 people each year in the UK, making it the fourth most common cancer among both men and women [1,2]. Globally, colorectal cancer is a leading cause of cancer-related deaths, ranking second only to lung cancer, with an estimated 904,000 deaths worldwide in 2022 [3]. Stage at diagnosis influences patient survival, with advanced disease having fewer treatment options and significantly worse outcomes. The 5-year survival rate for colorectal cancer in the UK is approximately 90% at the earliest stage (stage 1) but drops to just 10% at the most advanced stage (stage 4) [4].

Screening programs for colorectal cancer are well-established in the UK and aim to detect precancerous or early-stage cancers in asymptomatic individuals when a wider range of treatment options are available. The effectiveness of colorectal cancer screening is well documented, with evidence regular screening reduces disease-specific mortality [5]. Approximately, 11% of colorectal cancers are currently diagnosed via screening in England and in 2021/2022, 42% of patients presented with ‘early-stage’ colorectal cancer, an encouraging increase from 35% in 2017/2018 [6]. This is likely due in part to increased screening coverage, which has steadily risen over the last decade [7,8].

There are separate colorectal cancer screening programs for the different UK nations. England is currently in the process of lowering the screening age to 50 years. At present, individuals aged 54–74 years (50–74 in Scotland and Wales and 60–74 in Northern Ireland) are invited to participate in screening every two years using the fecal immunochemical test (FIT) – in 2019, FIT replaced the guaiac fecal occult blood test (gFOBT) in England, offering enhanced sensitivity and increased participant uptake [9–11]. Unlike gFOBT, FIT requires only a single stool sample, has no prior dietary restrictions and is specifically designed to detect human blood. Additionally, it provides a quantitative measure of fecal hemoglobin (f-HB) concentration, allowing screening programs to set their own thresholds for further testing. Currently, the threshold is 120 µg Hb/g in England and Wales, 80 µg Hb/g in Scotland and 150 µg Hb/g feces in Northern Ireland, with pilot sites across England starting to reduce to 80 µg Hb/g.

There is growing international enthusiasm for adopting a stratified approach to colorectal cancer screening, in which screening intensity and frequency would be tailored to an individual’s own risk of developing the disease [12,13]. Currently, age is the sole factor used to determine screening eligibility for individuals without a strong family history or certain high-risk conditions, such as Lynch syndrome. This approach has resulted in a screening framework based on the assumption of a uniform level of risk. However, colorectal cancer is influenced by both nonmodifiable risk factors (such as age, sex and genetics), and modifiable environmental and lifestyle factors [14]. Although age is the most significant determinant, a family history of colorectal cancer, along with lifestyle factors such as diet, alcohol consumption, and smoking, can all contribute to an individual’s risk. Stratified colorectal cancer screening has the potential to utilize these individual-specific risk factors along with quantitative f-HB levels, enabling a more personalized approach to screening adapted to each person’s unique risk profile.

At present, there are limited examples of risk-stratified colorectal cancer screening programs around the world and key uncertainties still exist around the feasibility and practicality of its implementation [15]. In this paper, we explore the current body of evidence on risk-stratified colorectal cancer screening and the potential impact of its implementation in the UK and globally incorporating ethical considerations and areas for further research. While this is not a systematic/formal narrative review, we identified literature describing risk-based bowel cancer screening by searching MEDLINE, EMBASE, and GOOGLE SCHOLAR. Key words searched included ‘colorectal cancer’. A manual search of reference lists of relevant studies was also conducted.

## 2. Stratified colorectal cancer screening – what strategies are possible?

Stratified colorectal cancer screening involves adjusting the intensity of screening based on an individual’s risk, rather than a ‘one size fits all’ approach. It enables targeting of screening to those most likely to benefit while reducing screening for low-risk individuals. This in turn can reduce screening-related harms, with evidence from modeling studies demonstrating reduced bowel perforations by 9.9% and a reduction in false positives by more than 48.6% using a stratified approach [12]. Risk stratification can be undertaken at various stages along the screening pathway; it can be used to determine individual eligibility, establish thresholds for further investigation and set intervals for follow up screening [16]. Two methods of interest to calculate individual risk, enabling risk-stratification across different aspects of the screening pathway are:

1. use personal bowel cancer risk (PBCR) information alone or in combination with FIT, to determine who and when to screen. This could be achieved in two ways.
  - use available modifiable and non-modifiable risk factor information such as age, sex, family history or lifestyle factors.
  - use personal genetic information in the form of polygenic risk scores.
2. use an individual’s quantitative FIT level from a previous screening episode to determine further investigation and follow up screening frequency.

### 2.1. Personal bowel cancer risk information to inform who, when and how to screen

PBCR information, based on a range of potential parameters, including age, sex, previous FIT screening results, genetics (polygenic risk scores, PGS), familial and lifestyle factors have the potential to refine and improve risk stratification beyond fHb level alone or age- and sex-specific FIT thresholds. Understanding which patients would benefit from earlier or more frequent screening or those requiring later or less frequent screening based on their individual risk factors could optimize screening, directing effort toward those whose increased risk means they stand to gain the most benefit.

Several risk prediction models have been designed to identify individuals at an elevated risk of developing colorectal cancer, by incorporating both modifiable and non-modifiable risk factors, such as family history and smoking status [17–24]. Utilizing this personal information has proven to be an effective method for risk stratification and has demonstrated improved detection of advanced colorectal neoplasia when combined with FIT [12,24,25]. This has the added advantage of using existing routine healthcare data, making implementation potentially less complex. There are still, however, significant challenges with incorporating individual risk factors alongside FIT, most notably around the collection and accuracy of personal data as many of the risk factors are not currently captured within GP records.

Recent advances in genome-wide association studies (GWASs) have identified hundreds of common-variant associations for colorectal cancer. PGS reflect individuals’ inherited susceptibility to disease by combining information from all the variants into a number or ‘personal risk’ [26]. There is growing interest in utilizing PGS to identify individuals at higher risk of developing colorectal cancer who may benefit from earlier or more frequent screening [27,28]. Integrating genetic information with preexisting risk prediction models has been demonstrated to improve the discrimination accuracy to colorectal cancer [29,30]. Recent studies evaluating the role of PGS in stratified bowel cancer screening are promising but the impact on health outcomes and resource use is yet to be fully examined in ‘real world’ settings and the benefits are likely to be seen most when PGS is used to identify those at higher risk who would benefit from earlier screening than when combined with a FIT screening result [31–34]. Ethical and economic implications of utilizing PGS in the general population also requires further exploration [12,35,36]. Genetic testing may be expensive and difficult to implement at a population level.

There is, however, strong interest in the UK in leveraging genetic and lifestyle information on a scale which can influence prevention and treatment programs at a population level across various health conditions, extending beyond colorectal cancer. If PGS is employed for other conditions, extending its application to colorectal cancer screening could enhance efficiency, potentially addressing some of the cost-related concerns. Notably, trials are underway incorporating (1) genetic and lifestyle information to risk stratify breast cancer screening, (2) lifestyle factors for lung cancer screening and (3) PGS-modified PSA testing in prostate cancer [37–45]. Further, programs such as Our Future Health are collecting genetic and lifestyle information from the population on a large scale [46]. This may provide information that could be used in the future to risk-stratify cancer screening programs.

### 2.2. Utilizing quantitative FIT levels to determine further investigation and follow-up screening

Currently, colorectal cancer screening across the UK uses a single ‘cut-off’ to identify individuals with a ‘positive’ test result who need further investigation. Individuals with a result above the threshold will be invited for further investigation such as a colonoscopy. All other individuals will be returned to routine screening every two years regardless of their actual level. This means that, in England, an individual with a result of 5 µg/g and an individual with 115 µg/g will return to the same screening regimen. Just over 50% of colorectal cancers are likely to be missed using a f-HB threshold of 120 µg/g [47]. Lowering the threshold will increase the sensitivity of testing but will lead to additional strain on colonoscopy resources.

Numerous studies have demonstrated a significant correlation between f-HB concentrations detected through FIT-based screening and the likelihood of colorectal cancer or significant adenomas, with higher concentrations seen in patients with more advanced disease [48–51]. Higher pre-threshold f-HB levels are also associated with greater risk of developing interval colorectal cancer (cancers diagnosed between routine screening), providing further evidence supporting a more stratified screening approach [52,53]. Leveraging the full range of f-HB concentrations available through FIT testing could potentially enable more targeted screening, without overwhelming colonoscopy capacity. There are various ways to fully leverage the full range of FIT results. One potential option is repeat testing or a reduced screening interval to those with ‘pre-threshold’ f-HB levels. Another option is to have longer screening intervals for those with very low/undetectable f-HB levels (levels below 10 µg/g carry an extremely low risk of developing interval cancers) [54].

These options could reduce unnecessary screening in those with very low risk, potentially saving resources, while increasing testing in individuals with higher risk who would benefit most from enhanced screening. Implementing such a stratified approach would, of course, require large organizational and structural changes. However, there is precedent for this in other screening programs, such as abdominal aortic aneurysm (AAA) screening for men aged 65 and cervical cancer screening for women in the UK, where follow-up frequency is based on the initial screening result.

Another area of growing interest is the adoption of sex and age-specific FIT ‘cut-offs’ whereby the threshold for further investigation is different between sexes or in different age categories [55,56]. Men for example have a higher risk of developing colorectal cancer than women and risk increases with age [55–57]. As such an older male with a pre-threshold FIT level might have a higher absolute risk of colorectal cancer on colonoscopy than a younger female. Age and sex are already available within screening records making this approach potentially easier to implement.

## 3. Ethical considerations to stratified screening

The UK National Screening Committee (UKNSC) describes four broad ethical principles which should be applied to screening including (1) improving health and wellbeing, (2) treating people with respect, (3) promoting equality and inclusion and (4) using public resources fairly and proportionately.

It is important to evaluate and consider how stratified screening addresses these principles before implementation.

### 3.1. Improving health and wellbeing

A targeted approach to stratified screening could improve the benefit-to-harm ratio by enhancing screening for individuals at higher risk, where the benefits are more significant, while reducing screening for those at lower risk, minimizing the potential for screening-related harms.

Providing individuals with information about their risk of developing cancer can have potential psychological implications. Information on individual risk may alleviate or worsen anxiety and there have been concerns from healthcare providers that individuals may disengage from screening or become overwhelmed with information [58]. However, a systematic review of the general public’s perspective on risk stratified cancer screening found a high desire for this information, describing knowledge of personalized cancer risk ‘empowering’ with high-risk individuals benefiting from the ‘safety net’ of increased monitoring and earlier intervention [59]. Existing evidence indicates no difference or in fact a reduction in cancer specific worry, anxiety or fear after receiving risk specific information [60,61]. The implications of providing the public with their f-HB level however have not yet been explored and this may have unique psychological consequences which will need to be reviewed.

### 3.2. Treating people with respect

Respecting individual autonomy and avoidance of coercion is vital in all screening programs [36]. Freedom to opt in or out of screening should not be compromised by the provision of risk information – individual’s must retain the right to decide based on their own personal preferences. Therefore, it is important individuals who do not wish to provide personal risk information can still access screening and no barriers are created by the addition of a risk-stratified approach.

### 3.3. Promoting equality and inclusion

It is crucial to examine any potential impact of implementing stratified colorectal cancer screening on health inequities and this is a major consideration among those involved in screening policy and service delivery. Despite the proven effectiveness of the current bowel screening program not all eligible groups participate equally. Several factors contribute to disparities in uptake including levels of deprivation and ethnicity [62,63]. Moving to risk-stratified screening might exacerbate these existing inequities further, deterring already marginalized groups. For example, providing personal risk information may be deemed less acceptable to certain groups, or more difficult to capture in deprived or ethnic minority groups. Language spoken at home has been demonstrated to significantly impact uptake of genomic testing for colorectal cancer risk assessment adding weight to these concerns [64]. Alternatively, risk stratification could help mitigate disparities, as many of the risk factors such as smoking are socially patterned. Incorporating these factors into screening strategies could shift resources to those who are most in need, including underserved communities. Currently, there is little existing evidence, and none for colorectal cancer specifically, on the impact in either direction, so it is vital this is explored in ‘real-world’ settings before any change in practice.

Health literacy is an independent predictor of screening adherence [65]. The increasing complexity of stratified screening programs may pose a further challenge for individuals with low health literacy, potentially leading to disengagement with screening [36].

There is also concern that PGS itself may exacerbate health disparities [66]. PGS has been unable to predict risk among non-European populations as accurately as European populations, because individuals in GWAS were primarily of European descent. Its validity was therefore much more limited in individuals of African, Latin American and Asian ancestry [66,67]. There has been recent strives in GWAS toward increasing the diversity of populations included to improve the applicability of PGS across different populations in recent years and this may become less of a concern as advances in GWAS continue [68].

### 3.4. Using public resources fairly and proportionately

Bowel cancer screening is already in place within the UK and in many other countries around the world. Stratified screening could enhance the existing screening protocol to result in better use of public resources, by targeting those most in need. This is particularly relevant in a National Health Service where the allocation of resources must be carefully considered and any decisions regarding screening should have robust evidence from cost-effectiveness analyses. Particularly as resources such as colonoscopy are constrained. However, a stratified approach using PBCR has been shown in a modeling study to significantly reduce the colonoscopy load by over 90% with a corresponding higher positive predictive value for any neoplasm [69]. Therefore, stratified screening may reduce the demand on colonoscopy’s but this needs to be weighed against the cost of setting up and completing the risk assessments.

## 4. Public acceptability

Acceptability and potential impact on uptake are key factors to consider ahead of implementation of any risk-based approaches to colorectal cancer screening and the UKNSC guidance necessitates the acceptability of screening programs from both social and ethical perspectives [69–71]. Public acceptability is a necessary component of interventions for colorectal cancer screening as it will likely have implications for uptake, which determines screening program outcomes and ultimately feasibility of the approach [72,73]. Furthermore, sufficient understanding of risk-stratified screening, a fundamental component of acceptability, is required to ensure informed decision-making and consent to participate among members of the public [74].

Evidence suggests that applying a personalized approach to cancer screening in general is highly acceptable [59]. This holds true in the case of colorectal cancer screening specifically, where public acceptability of stratified screening incorporating data on PBCR, has previously been explored using a range of approaches [16,72,75,76]. A 2023 community jury study exploring the implementation of different risk-stratified strategies for colorectal cancer screening from a societal perspective reported strong public acceptability of such an approach, with some participants considering this to be an obvious change to the screening program in which the benefits outweighed potential concerns, including the implications of de-intensified screening for low-risk individuals [16]. Further research has demonstrated that the public largely find stratified bowel screening to align with their preferences and values, with priority given to the number of lives saved and maintaining/improving program sensitivity [75,76]. Nevertheless, de-intensification of screening among low-risk individuals is generally considered less acceptable than increasing screening for those at higher risk [59,72].

Although there is a paucity of data on how stratification of the colorectal cancer screening program might impact uptake, there is some evidence to suggest that a risk-based approach may motivate screening attendance among high-risk groups and improve uptake in average-risk groups – however, there is also the potential that, at the individual level, low-risk individuals may be disincentivized from completing screening, possibly owing to a false sense of reassurance as a result of their low-risk status [72,77,78]. A recent survey study reporting anticipated uptake of risk-stratified FIT screening at different risk levels, found that 16.8% fewer participants stated that they would take up screening if offered a low-risk screening protocol than they would if offered age-based screening [72]. Implications for FIT uptake remain an important consideration prior to implementing stratified screening and greater evidence on the perspective of low-risk individuals and how to communicate low risk in practice is required.

## 5. Would it be cost-effective?

Cost-effectiveness is a major driver in stratified approaches to cancer screening, enabling resources to be directed toward high benefit individuals, while reducing activity in lower-risk individuals. Resource constraints for NHS cancer screening programs will be an ongoing feature in the years ahead, exacerbated by the COVID-19 pandemic [79]. Three studies have involved micro-simulated cost-effectiveness analyses of colorectal cancer screening informed by PGS in England, Australia, and the US [27,35,80,81]. Findings from a UK-based health economic analysis suggested that a personalized, risk-stratified approach to determine screening eligibility could be cost-effective compared to inviting all individuals for screening at age 60 [27]. In contrast, the other studies concluded that PGS-informed colorectal cancer screening, whether combined with FIT or colonoscopy, was unlikely to be cost-effective, primarily due to the high cost of determining individual risk [35,80]. However, if the cost of risk determination were reduced, this approach might become more cost-effective and lead to fewer colonoscopies [35].

With this mixed picture, further research is needed to evaluate the cost-effectiveness of risk-stratified approaches to colorectal cancer screening in ‘real word’ settings [13]. This evidence is crucial to inform policy decisions and ensure that such approaches improve both clinical and health-economic outcomes.

## 6. Current status of UK colorectal cancer screening and future priorities

At present, there is significant change occurring within NHS colorectal cancer screening. All screening programs face significant post-Covid 19 challenges; endoscopy provision is stretched, and there is significant ‘catch-up’ demand on diagnostic services, as Covid-19 led to many people with symptoms deferring presentation [79]. There are also trials of lowering age and FIT thresholds in England currently underway. Within this constrained NHS environment transitioning to risk-stratified colorectal cancer screening would have substantial organizational implications, with adjustments required to screening pathways, IT infrastructure, and quality assurance protocols [82]. Additionally, robust safeguards would need to be established to address ethical, legal, and privacy concerns. At present, we do not know how feasible these changes would be; indeed, the most ‘basic’ change (such as changing a ‘cut-off’ FIT level), requires careful planning prior to implementation. Further, feasibility of screening will vary across the UK, according to local context and organizational characteristics. Nevertheless, within interviews in which organizational feasibility was prominent, stakeholders involved in delivering the screening program in England considered that risk-stratification would be beneficial to the current screening program [82]. It is vital, therefore, that any move toward stratified screening works around these current priorities. We suggest that some aspects, such as using the full range of f-HB levels to drive screening regimens could be implemented without significant disruption and cost. While incorporating genetic and lifestyle information is more complex, we believe this information will become more readily available and accessible over the next decade, enhancing the prospect of screening regimens driven by multi-factorial risk models. Accordingly, ‘real world’ evidence is required to guide any transition to risk-stratified approaches. Potential benefits and ‘downsides’ of this transition are shown in Table 1.

**Table 1.** Potential benefits and downsides of stratified colorectal cancer screening.

Potential benefits	Potential downsides
More efficient resource allocation	Ethical concerns
More frequent screening for high-risk individuals	Worsening of health inequalities
Earlier cancer detection and decreased cancer incidence	Increases in cost
Better use of the full range of quantitative FIT results	Significant organizational/IT changes required and complex implementation
Reduction in screening-related harms	Availability and reliability of data on personal risk factors
Addresses NHS priority for more individualized prevention and treatment	Data privacy and consent
Increased compliance in high-risk individuals	Possible decreased compliance in low-risk individuals
‘Empowering’ effect of personal risk knowledge	Worsening of health anxiety

FIT: fecal immunochemical test; IT: information technology.

## 7. The research agenda: what do we need to know before adopting stratified approaches in UK colorectal cancer screening?

At present, we lack the evidence to recommend a change to risk-stratified colorectal cancer screening in the UK. In Table 2, we list a number of areas where further research is needed. Importantly, building on existing data from hypothetical scenarios and modeling studies, we now need evidence from studies conducted in ‘real-world’ settings – that is, we need to pilot risk-stratified approaches within existing NHS bowel cancer screening programs. In the absence of these ‘real-world’ data, our ability to validate existing models, examine acceptability, and explore impact on key outcomes such as organizational impact and cost-effectiveness, is limited. To this end, Cancer Research UK has recently funded a program of work in which NHS bowel screening participants will, for the first time, be offered risk-stratified options [83]. The aim of this work is to provide the NHS with the information needed in order to transition toward risk-stratification within its bowel screening programs.

**Table 2.** Proposed research agenda for risk-based colorectal cancer screening implementation in the UK.

Evidence gap	Research recommendation	Proposed approach/methods
‘Real world’ data from f-HB-driven risk-based screening	Drawing on best-available evidence we need to establish cohort studies within existing NHS programs in which participants are offered alternative screening regimens based on their actual f-HB level, rather than the current binary (‘positive’ vs. ‘negative’) approach	Recruitment from current screening participants, establish a ‘pre-threshold’ interval with a changed screening regimen – possibly repeat testing, or reduced screening interval
‘Real-world’ evidence on stratifying screening based on personal bowel cancer risk (derived from genetic/lifestyle/family history information)	We need to explore the feasibility of collecting this information in screening participants – and to examine prospectively how using PBCR information might impact on screening outcomes	Pilot methods of collecting PBCR information – through questionnaires (paper or digital), explore options for obtaining information from existing sources – e.g., primary care records, population-based initiatives collecting PBCR information
Acceptability of risk-based screening based on real-world data (current evidence based on hypothetic scenarios)	Descriptive studies in which acceptability is examined among bowel screening participants who are either offered risk-based approaches or in whom PBCR information is collected. This needs to include acceptability of offering less screening to lower-risk individuals	Mixed methods approaches in groups offered risk-based screening strategies – combining qualitative and quantitative methods. Ideally using validated measures of acceptability
Health economic models based on inputs from ‘real world’ settings	Existing health economic models need to be refined and updated with data from ‘real world’ settings in which participants have experienced screening regimens which have been risk-adapted. Refined models should ideally incorporate risk from both quantitative FIT and PBCR data	Micro-simulation modeling is potentially the best approach. It has the capacity to handle the large amounts of data in screening data-sets, model individual behaviors within a screening program, and analyze the impact of different screening regimens
Impact on health inequalities (at present we only have theoretical understanding)	Studies where uptake in risk-stratified screening is analyzed by factors such as deprivation and ethnicity. Qualitative studies focusing on deprived and ethnic groups	Much could be determined through screening program datasets – although capturing ethnicity data remains a challenge. Sophisticated recruitment techniques required to capture views of non-participants in descriptive studies
Impact on current NHS screening programs	Studies in which impact (of changes required for risk-stratified approaches) on IT and organizational structures is examined	Routine activity data from screening centers and hubs, coupled with descriptive/qualitative surveys of key stakeholders, including screening personnel

FIT: fecal immunochemical test; f-HB: fecal hemoglobin; PBCR: personal bowel cancer risk information.

## 8. Conclusions

Screening has led to reductions in incidence and mortality from colorectal cancer, but there is significant potential to improve current early diagnosis and screening efforts. Moving to risk-based/stratified approaches to colorectal cancer screening offers the prospect of improved screening outcomes, achieved without significant extra costs. Existing evidence in areas including colorectal cancer risk-modeling, cost-effectiveness and acceptability suggest that transitioning to risk-based screening should be considered seriously by UK colorectal cancer screening programs. In order to do this, we need evidence from studies in which risk-based strategies are piloted within current screening programs. As disease prevention and management moves toward more personalized approaches around the world, it is important that colorectal cancer screening programs fully explore and understand these approaches, in the hope of refining their programs and reducing the burden of this disease.

## 9. Future perspective

This is a rapidly changing field. There is an expectation that, over the next few years, risk information of various types will be more readily available – to a growing proportion of the population. Current initiatives such as Our Future Health, and the advent of multi-cancer early detection tests highlight this trend.

- we predict that in 5–10 years’ time all screening programs will routinely incorporate risk information. This information will be collected from routinely available health data – with much of the burden of collection alleviated by artificial intelligence approaches.
- not only will risk information be more available – we will become more adept at using this information to tailor diagnostic testing, screening and treatment.
- in the case of bowel cancer screening, we believe that FIT will still be in use, but instead of acting on arbitrary thresholds we will use the actual f-HB result to inform decisions.
- it is possible that in the future there will be additional tests – based on technologies such as circulating DNA. We will have more robust and repeatable models to combine risk information from all these various sources.
- There will be an increased awareness of risk literacy within the community, with people gaining a clearer understanding of the potential and limitations of this information. Additionally, there will be a stronger collective understanding of the ethical issues associated with it.
- of course, capacity and resources will continue to shape screening services; it is possible that techniques such as capsule endoscopy will have improved by the end of the next decade, offering greater accessibility to diagnostic investigations.

## 10. Article highlights

### 10.1. Introduction

- Colorectal cancer is a leading cause of cancer-related deaths globally.
- Screening programs using FIT are well established in the UK.
- Eleven percent of colorectal cancers are diagnosed via screening in England.
- At present, age is the sole factor used to stratify screening in the UK – individuals between 50 and 74 are invited for screening every two years (starting age varies across the UK nations).

### 10.2. Stratified colorectal cancer screening – what strategies are possible?

- Stratified colorectal cancer screening adjusts the intensity of screening based on an individual’s risk – either based on test results and/or personal risk factors.
- FIT provides a quantitative measure of f-HB which can be used to determine screening need for further diagnostic tests or screening frequency.
- Personal bowel cancer risk can help identify individuals at higher risk of developing disease who may benefit from enhanced screening.
- PBCR may include age, sex, genetics and lifestyle factors alongside previous FIT results.
- The feasibility of these various strategies remains uncertain – changing screening regimens is not straightforward, and collection/incorporation of genetic lifestyle information can be complex – we need feasibility evidence before moving to risk-based approaches.

### 10.3. Ethical considerations to stratified screening

- Stratified screening may exacerbate existing inequalities in screening uptake through disparities in genetic risk profiling, health literacy and potentially marginalizing underrepresented groups.
- Providing individual cancer risk information may have psychological implications but current research suggests the public welcome this knowledge.
- Individual autonomy should be at the forefront of implementation of stratified screening.

### 10.4. Public acceptability

- Acceptability is a key consideration prior to implementation of stratified screening.
- Current evidence suggests a personalized approach to cancer screening is highly acceptable to the public – but further data are needed following real-world implementation.
- There is a lack of data on how stratified screening may impact on uptake, particularly in ‘low risk individuals’.

### 10.5. Would it be cost-effective?

- Current evidence on the cost-effectiveness of stratified bowel screening is mixed and needs to be strengthened based on real-world data to better inform policy decisions.

### 10.6. Current status of UK colorectal cancer screening and future priorities

- The NHS is facing significant post-Covid 19 challenges, with stretched resources and a significant ‘catch-up’ demand on diagnostic services.
- Transitioning to risk-stratified colorectal cancer screening would have substantial organizational implications, with adjustments required to screening pathways, IT infrastructure, and quality assurance protocols.

### 10.7. The research agenda: what do we need to know before adopting stratified approaches in UK colorectal cancer screening

- Most of the existing data are based on hypothetical scenarios and modeling studies.
- Pilots of risk-stratified approaches are needed to fully understand the clinical, operational and economic implications of such strategies in practice.
- Cancer Research UK is funding a program of work to produce evidence required to underpin a transition to risk-stratified bowel cancer screening in the UK.

### 10.8. Conclusions

- Stratified screening has the potential to improve screening outcomes without significant extra costs.
- Further research is needed to fully explore the implications of stratified screening particularly in real world settings.

## References

[1] Bowel Research UK. Bowel cancer; 2024 [accessed 2024 Nov 1]. Available from: https://www.bowelresearchuk.org/about-bowels/bowel-cancer/#:∼:text=Bowel%20cancer%20statistics%20In%20the%20UK%3A%2042%2C000%20new,largest%20cause%20of%20cancer%20deaths%20%28behind%20lung%20cancer%29

[2] IARC. International Agency for Research on Cancer. Globocan 2018: cancer fact sheets – colorectal cancer; 2018 [accessed 2024 Nov 10]. Available from: http://gco.iarc.fr/today/data/factsheets/cancers/10_8_9-Colorectum-fact-sheet.pdf

[3] Bray F, Laversanne M, Sung H, et al. Global cancer statistics 2022: GLOBOCAN estimates of incidence and mortality worldwide for 36 cancers in 185 countries. CA Cancer J Clin. 2024;74(3):229–263. doi: 10.3322/caac.21763 38572751

[4] UK screening and surveillance for bowel cancers. StatPearls; Tampa, Florida 2024. 38261693

[5] Gini A, Jansen EEL, Zielonke N, et al. Impact of colorectal cancer screening on cancer-specific mortality in Europe: a systematic review. Eur J Cancer. 2020;127:224–235. doi: 10.1016/j.ejca.2019.12.035 31932176

[6] Routes to diagnosis dashboard: incidence [Internet]; 2024 [accessed 2024 Nov 1]. Available from: https://nhsd-ndrs.shinyapps.io/routes_to_diagnosis/

[7] National Bowel Cancer Audit. State of the nation report. National Bowel Cancer Audit; 2024 [accessed 2024 Nov 10]. Available from: https://www.nboca.org.uk/reports/state-of-the-nation-report/

[8] NHS England. Bowel cancer screening annual report 2021 to 2022; 2024 [accessed 2024 Nov 10]. Available from: https://www.gov.uk/government/publications/bowel-cancer-screening-annual-report-2021-to-2022/bowel-cancer-screening-annual-report-2021-to-2022

[9] Mankaney G, Sutton RA, Burke CA. Colorectal cancer screening: choosing the right test. Cleve Clin J Med. 2019;86(6):385–392. doi: 10.3949/ccjm.86a.17125 31204977

[10] Mousavinezhad M, Majdzadeh R, Akbari Sari A, et al. The effectiveness of FOBT vs. FIT: a meta-analysis on colorectal cancer screening test. Med J Islam Repub Iran. 2016;30:366. 27493910 PMC4972062

[11] Moss S, Mathews C, Day TJ, et al. Increased uptake and improved outcomes of bowel cancer screening with a faecal immunochemical test: results from a pilot study within the National Screening Programme in England. Gut. 2017;66(9):1631–1644. doi: 10.1136/gutjnl-2015-310691 27267903

[12] Cairns JM, Greenley S, Bamidele O, et al. A scoping review of risk-stratified bowel screening: current evidence, future directions. Cancer Causes Control. 2022;33(5):653–685. doi: 10.1007/s10552-022-01586-8• This is a scooping review of the international literature on risk-stratified bowel screening. It provides a comprehensive overview of the current research landscape, with sections on diagnostic performance, effectiveness, acceptability and cost-effectiveness of stratified screening. 35306592 PMC8934381

[13] Hull MA, Rees CJ, Sharp L, et al. A risk-stratified approach to colorectal cancer prevention and diagnosis. Nat Rev Gastroenterol Hepatol. 2020;17(12):773–780. doi: 10.1038/s41575-020-00368-3 33067592 PMC7562765

[14] Kanth P, Inadomi JM. Screening and prevention of colorectal cancer. BMJ. 2021;374:n1855. doi: 10.1136/bmj.n1855 34526356

[15] Lansdorp-Vogelaar I, Meester R, de Jonge L, et al. Risk-stratified strategies in population screening for colorectal cancer. Int J Cancer. 2022;150(3):397–405. doi: 10.1002/ijc.33784 34460107 PMC9293115

[16] Taylor LC, Dennison RA, Griffin SJ, et al. Implementation of risk stratification within bowel cancer screening: a community jury study exploring public acceptability and communication needs. BMC Public Health. 2023;23(1):1798. • This study describes the results of two community juries exploring risk stratification eligibility, thresholds for referral and screening intervals and demonstrate that to an informed public risk stratified screening was not only acceptable but considered an obvious next step. doi: 10.1186/s12889-023-16704-6 37715213 PMC10503141

[17] Ma GK, Ladabaum U. Personalizing colorectal cancer screening: a systematic review of models to predict risk of colorectal neoplasia. Clin Gastroenterol Hepatol. 2014;12(10):1624–1634.e1. doi: 10.1016/j.cgh.2014.01.042 24534546

[18] Cai Q-C, Yu E-D, Xiao Y, et al. Derivation and validation of a prediction rule for estimating advanced colorectal neoplasm risk in average-risk Chinese. Am J Epidemiol. 2012;175(6):584–593. doi: 10.1093/aje/kwr337 22328705

[19] Ma E, Sasazuki S, Iwasaki M, et al. 10-Year risk of colorectal cancer: development and validation of a prediction model in middle-aged Japanese men. Cancer Epidemiol. 2010;34(5):534–541. doi: 10.1016/j.canep.2010.04.021 20554262

[20] Freedman AN, Slattery ML, Ballard-Barbash R, et al. Colorectal cancer risk prediction tool for white men and women without known susceptibility. J Clin Oncol. 2009;27(5):686–693. doi: 10.1200/JCO.2008.17.4797 19114701 PMC2645090

[21] Walker JG, Bickerstaffe A, Hewabandu N, et al. The CRISP colorectal cancer risk prediction tool: an exploratory study using simulated consultations in Australian primary care. BMC Med Inform Decis Mak. 2017;17(1):13. doi: 10.1186/s12911-017-0407-7 28103848 PMC5248518

[22] Nartowt BJ, Hart GR, Roffman DA, et al. Scoring colorectal cancer risk with an artificial neural network based on self-reportable personal health data. PLOS One. 2019;14(8):e0221421. doi: 10.1371/journal.pone.0221421 31437221 PMC6705772

[23] Hippisley-Cox J, Coupland C. Development and validation of risk prediction algorithms to estimate future risk of common cancers in men and women: prospective cohort study. BMJ Open. 2015;5(3):e007825. doi: 10.1136/bmjopen-2015-007825 PMC4368998 25783428

[24] Smith T, Muller DC, Moons KGM, et al. Comparison of prognostic models to predict the occurrence of colorectal cancer in asymptomatic individuals: a systematic literature review and external validation in the EPIC and UK Biobank prospective cohort studies. Gut. 2019;68(4):672–683. doi: 10.1136/gutjnl-2017-315730 29615487 PMC6580880

[25] Cooper JA, Ryan R, Parsons N, et al. The use of electronic healthcare records for colorectal cancer screening referral decisions and risk prediction model development. BMC Gastroenterol. 2020;20(1):78. doi: 10.1186/s12876-020-01206-1 32213167 PMC7093989

[26] Sugrue LP, Desikan RS. What are polygenic scores and why are they important? JAMA. 2019;321(18):1820–1821. doi: 10.1001/jama.2019.3893 30958510

[27] Thomas M, Sakoda LC, Hoffmeister M, et al. Genome-wide modeling of polygenic risk score in colorectal cancer risk. Am J Hum Genet. 2020;107(3):432–444. doi: 10.1016/j.ajhg.2020.07.006 32758450 PMC7477007

[28] Obón-Santacana M, Díez-Villanueva A, Alonso MH, et al. Polygenic risk score across distinct colorectal cancer screening outcomes: from premalignant polyps to colorectal cancer. BMC Med. 2021;19(1):261. doi: 10.1186/s12916-021-02134-x 34743725 PMC8574048

[29] Briggs SEW, Law P, East JE, et al. Integrating genome-wide polygenic risk scores and non-genetic risk to predict colorectal cancer diagnosis using UK Biobank Data: population based cohort study. BMJ. 2022;379:e071707. doi: 10.1136/bmj-2022-071707 36351667 PMC9644277

[30] Sassano M, Mariani M, Quaranta G, et al. Polygenic risk prediction models for colorectal cancer: a systematic review. BMC Cancer. 2022;22(1):65. doi: 10.1186/s12885-021-09143-2 35030997 PMC8760647

[31] Tamlander M, Jermy B, Seppälä TT, et al. Genome-wide polygenic risk scores for colorectal cancer have implications for risk-based screening. Br J Cancer. 2024;130(4):651–659. doi: 10.1038/s41416-023-02536-z 38172535 PMC10876651

[32] Guo F, Edelmann D, Cardoso R, et al. Polygenic risk score for defining personalized surveillance intervals after adenoma detection and removal at colonoscopy. Clin Gastroenterol Hepatol. 2023;21(1):210–219.e11. doi: 10.1016/j.cgh.2022.03.013 35331942

[33] Guo F, Weigl K, Carr PR, et al. Use of polygenic risk scores to select screening intervals after negative findings from colonoscopy. Clin Gastroenterol Hepatol. 2020;18(12):2742–2751.e7. doi: 10.1016/j.cgh.2020.04.077 32376506

[34] Chen H, Liu L, Lu M, et al. Implications of lifestyle factors and polygenic risk score for absolute risk prediction of colorectal neoplasm and risk-adapted screening. Front Mol Biosci. 2021;8:685410. doi: 10.3389/fmolb.2021.685410 34336927 PMC8324207

[35] Naber SK, Kundu S, Kuntz KM, et al. Cost-effectiveness of risk-stratified colorectal cancer screening based on polygenic risk: current status and future potential. JNCI Cancer Spectr. 2020;4(1):pkz086. doi: 10.1093/jncics/pkz086 32025627 PMC6988584

[36] Dennison RA, Usher-Smith JA, John SD. The ethics of risk-stratified cancer screening. Eur J Cancer. 2023;187:1–6. • This is an interesting opinion article on the ethical considerations of risk-stratified cancer screening. Particularly how risk stratified screening falls within the principles of medical ethics including beneficence, non-maleficence, justice and respect for autonomy. doi: 10.1016/j.ejca.2023.03.023 37094523

[37] Clift AK, Dodwell D, Lord S, et al. The current status of risk-stratified breast screening. Br J Cancer. 2022;126(4):533–550. doi: 10.1038/s41416-021-01550-3 34703006 PMC8854575

[38] French DP, Astley S, Brentnall AR, et al. What are the benefits and harms of risk stratified screening as part of the NHS Breast Screening Programme? Study protocol for a multi-site non-randomised comparison of BC-predict versus usual screening (NCT04359420). BMC Cancer. 2020;20(1):570. doi: 10.1186/s12885-020-07054-2 32552763 PMC7302349

[39] Esserman L, Eklund M, Veer L, et al. The WISDOM study: a new approach to screening can and should be tested. Breast Cancer Res Treat. 2021;189(3):593–598. doi: 10.1007/s10549-021-06346-w 34529196

[40] Shieh Y, Eklund M, Madlensky L, et al. Breast cancer screening in the precision medicine era: risk-based screening in a population-based trial. J Natl Cancer Inst. 2017;109(5):djw290. doi: 10.1093/jnci/djw290 28130475

[41] Seung S-J, Mittmann N, Ante Z, et al. Evaluating real world health system resource utilization and costs for a risk-based breast cancer screening approach in the Canadian PERSPECTIVE Integration and Implementation Project. Cancers. 2024;16 18):3189. doi: 10.3390/cancers16183189 39335160 PMC11430316

[42] Osses DF, Roobol MJ, Schoots IG. Prediction medicine: biomarkers, risk calculators and magnetic resonance imaging as risk stratification tools in prostate cancer diagnosis. Int J Mol Sci. 2019;20(7):1637. doi: 10.3390/ijms20071637 30986955 PMC6480079

[43] Burki T. Prostate Cancer UK launches the TRANSFORM trial. Lancet. 2024;403(10438):1738. doi: 10.1016/S0140-6736(24)00912-7 38705155

[44] Davidson KW, Barry MJ, Mangione CM, et al. Screening for colorectal cancer: US Preventive Services Task Force Recommendation Statement. JAMA. 2021;325(19):1965–1977. doi: 10.1001/jama.2021.6238 34003218

[45] Balata H, Ruparel M, O’Dowd E, et al. Analysis of the baseline performance of five UK Lung Cancer Screening Programmes. Lung Cancer. 2021;161:136–140. doi: 10.1016/j.lungcan.2021.09.012 34583222

[46] UKRI UK Research and Innovation. Our Future Health becomes largest research programme of its kind; 2024 [accessed 2024 Nov 12]. Available from: https://www.ukri.org/news/our-future-health-becomes-largest-research-programme-of-its-kind/

[47] Li SJ, Sharples LD, Benton SC, et al. Faecal immunochemical testing in bowel cancer screening: estimating outcomes for different diagnostic policies. J Med Screen. 2021;28(3):277–285. doi: 10.1177/0969141320980501 33342370 PMC8366184

[48] Navarro M, Hijos G, Ramirez T, et al. Fecal hemoglobin concentration, a good predictor of risk of advanced colorectal neoplasia in symptomatic and asymptomatic patients. Front Med. 2019;6:91. doi: 10.3389/fmed.2019.00091 PMC6510055 31131279

[49] Liao CS, Lin YM, Chang HC, et al. Application of quantitative estimates of fecal hemoglobin concentration for risk prediction of colorectal neoplasia. World J Gastroenterol. 2013;19(45):8366–8372. doi: 10.3748/wjg.v19.i45.8366 24363529 PMC3857461

[50] Balamou C, Koïvogui A, Rodrigue CM, et al. Prediction of the severity of colorectal lesion by fecal hemoglobin concentration observed during previous test in the French Screening Program. World J Gastroenterol. 2021;27(31):5272–5287. doi: 10.3748/wjg.v27.i31.5272 34497450 PMC8384754

[51] Auge JM, Pellise M, Escudero JM, et al. Risk stratification for advanced colorectal neoplasia according to fecal hemoglobin concentration in a colorectal cancer screening program. Gastroenterology. 2014;147(3):628–636.e1. doi: 10.1053/j.gastro.2014.06.008 24937264

[52] Breekveldt ECH, Toes-Zoutendijk E, van de Schootbrugge-Vandermeer HJ, et al. Factors associated with interval colorectal cancer after negative FIT: results of two screening rounds in the Dutch FIT-based CRC Screening Program. Int J Cancer. 2023;152(8):1536–1546. • This is a study analyzing the Dutch colorectal cancer screening programme which involves biennially FIT-based screening. In this study, they analyze the characteristics of interval colorectal cancers and effectively illustrate increasing pre-threshold f-HB levels are associated with an increased risk of developing interval colorectal cancer and that individuals with unmeasurable f-HB levels have a corresponding very low risk. This lends support to the argument for risk stratified colorectal cancer screening utilizing the full range of f-HB. doi: 10.1002/ijc.34373 36444504 PMC10107864

[53] Toes-Zoutendijk E, Kooyker AI, Dekker E, et al. Incidence of interval colorectal cancer after negative results from first-round fecal immunochemical screening tests, by cutoff value and participant sex and age. Clin Gastroenterol Hepatol. 2020;18(7):1493–1500. doi: 10.1016/j.cgh.2019.08.021 31442598

[54] Grobbee EJ, Schreuders EH, Hansen BE, et al. Association between concentrations of hemoglobin determined by fecal immunochemical tests and long-term development of advanced colorectal neoplasia. Gastroenterology. 2017;153(5):1251–1259.e2. 2017/11/01/. doi: 10.1053/j.gastro.2017.07.034 28760383

[55] McDonald PJ, Strachan JA, Digby J, et al. Faecal haemoglobin concentrations by gender and age: implications for population-based screening for colorectal cancer. Clin Chem Lab Med. 2011;50(5):935–940. doi: 10.1515/CCLM.2011.815 22149740

[56] Kortlever TL, van der Vlugt M, Dekker E, et al. Individualized faecal immunochemical test cut-off based on age and sex in colorectal cancer screening. Prev Med Rep. 2021;23:101447. doi: 10.1016/j.pmedr.2021.101447 34168954 PMC8209662

[57] White A, Ironmonger L, Steele RJC, et al. A review of sex-related differences in colorectal cancer incidence, screening uptake, routes to diagnosis, cancer stage and survival in the UK. BMC Cancer. 2018;18(1):906. doi: 10.1186/s12885-018-4786-7 30236083 PMC6149054

[58] Hawkins R, McWilliams L, Ulph F, et al. Healthcare professionals’ views following implementation of risk stratification into a National Breast Cancer Screening Programme. BMC Cancer. 2022;22(1):1058. doi: 10.1186/s12885-022-10134-0 36224549 PMC9555254

[59] Taylor LC, Hutchinson A, Law K, et al. Acceptability of risk stratification within population-based cancer screening from the perspective of the general public: a mixed-methods systematic review. Health Expect. 2023;26(3):989–1008. doi: 10.1111/hex.13739 36852880 PMC10154794

[60] Bayne M, Fairey M, Silarova B, et al. Effect of interventions including provision of personalised cancer risk information on accuracy of risk perception and psychological responses: a systematic review and meta-analysis. Patient Educ Couns. 2020;103(1):83–95. • This systematic review summarizes the current evidence on the psychological impact of providing personalised cancer risk information at a population level. Most studies focused on personalised breast cancer risk; however, six studies involved colorectal cancer risk. This systematic review illustrates that most of the evidence indicates personal risk information does not worsen cancer related anxiety. doi: 10.1016/j.pec.2019.08.010 31439435 PMC6919334

[61] French DP, Southworth J, Howell A, et al. Psychological impact of providing women with personalised 10-year breast cancer risk estimates. Br J Cancer. 2018;118(12):1648–1657. doi: 10.1038/s41416-018-0069-y 29736008 PMC6008295

[62] Campbell C, Douglas A, Williams L, et al. Are there ethnic and religious variations in uptake of bowel cancer screening? A retrospective cohort study among 1.7 million people in Scotland. BMJ Open. 2020;10(10):e037011. doi: 10.1136/bmjopen-2020-037011 PMC7542953 33033017

[63] Quyn AJ, Fraser CG, Stanners G, et al. Uptake trends in the Scottish Bowel Screening Programme and the influences of age, sex, and deprivation. J Med Screen. 2018;25(1):24–31. doi: 10.1177/0969141317694065 29183246

[64] Saya S, McIntosh JG, Winship IM, et al. A genomic test for colorectal cancer risk: is this acceptable and feasible in primary care? Public Health Genomics. 2020;23(3–4):110–121. doi: 10.1159/000508963 32688362

[65] Baccolini V, Isonne C, Salerno C, et al. The association between adherence to cancer screening programs and health literacy: a systematic review and meta-analysis. Prev Med. 2022;155:106927. doi: 10.1016/j.ypmed.2021.106927 34954244

[66] Roberts MC, Khoury MJ, Mensah GA. Perspective: the clinical use of polygenic risk scores: race, ethnicity, and health disparities. Ethn Dis. 2019;29(3):513–516. doi: 10.18865/ed.29.3.513 31367172 PMC6645721

[67] Martin AR, Kanai M, Kamatani Y, et al. Clinical use of current polygenic risk scores may exacerbate health disparities. Nat Genet. 2019;51(4):584–591. doi: 10.1038/s41588-019-0379-x 30926966 PMC6563838

[68] Kachuri L, Chatterjee N, Hirbo J, et al. Principles and methods for transferring polygenic risk scores across global populations. Nat Rev Genet. 2024;25(1):8–25. doi: 10.1038/s41576-023-00637-2 37620596 PMC10961971

[69] Dyer TA, Owens J, Robinson PG. The acceptability of healthcare: from satisfaction to trust. Community Dent Health. 2016;33(4):242–251. doi: 10.1922/CDH_3902Dyer10 28537359

[70] GOV.UK. Criteria for appraising the viability, effectiveness and appropriateness of a screening programme; 2024 [accessed 2024 Nov 20]. Available from: https://www.gov.uk/government/publications/evidence-review-criteria-national-screening-programmes/criteria-for-appraising-the-viability-effectiveness-and-appropriateness-of-a-screening-programme

[71] NLeatherman S, Sutherland K. The quest for quality: refining the NHS reforms: a policy analysis and chartbook; 2008. 259 pp. Nuffield Trust, London

[72] Taylor LC, Dennison RA, Usher-Smith JA. Public acceptability and anticipated uptake of risk-stratified bowel cancer screening in the UK: an online survey. Prev Med Rep. 2024;48:102927. doi: 10.1016/j.pmedr.2024.102927 39634284 PMC11614824

[73] Sidani S, Miranda J, Epstein DR, et al. Relationships between personal beliefs and treatment acceptability, and preferences for behavioral treatments. Behav Res Ther. 2009;47(10):823–829. doi: 10.1016/j.brat.2009.06.009 19604500 PMC2742570

[74] Sekhon M, Cartwright M, Francis JJ. Acceptability of health care interventions: a theoretical framework and proposed research agenda. Br J Health Psychol. 2018;23(3):519–531. doi: 10.1111/bjhp.12295 29453791

[75] Dennison RA, Clune RJ, Morris S, et al. Understanding the preferences and considerations of the public towards risk-stratified screening for colorectal cancer: insights from think-aloud interviews based on a discrete choice experiment. Health Expect. 2024;27(4):e14153. doi: 10.1111/hex.14153 39030943 PMC11258464

[76] Dennison RA, Thomas CV, Morris S, et al. A discrete choice experiment to understand public preferences and priorities for risk-stratified bowel cancer screening programmes in the UK. Prev Med. 2023;177:107786. doi: 10.1016/j.ypmed.2023.107786 37984646

[77] Yen T, Qin F, Sundaram V, et al. Randomized controlled trial of personalized colorectal cancer risk assessment vs ­education to promote screening uptake. Am J Gastroenterol. 2021;116(2):391–400. doi: 10.14309/ajg.0000000000000963 33009045

[78] Emery JD, Jenkins MA, Saya S, et al. The Colorectal cancer RISk Prediction (CRISP) trial: a randomised controlled trial of a decision support tool for risk-stratified colorectal cancer screening. Br J Gen Pract. 2023;73(733):e556–e565. doi: 10.3399/BJGP.2022.0480 37012077 PMC10098832

[79] Armitage RC, Morling JR. The impact of COVID-19 on National Screening Programmes in England. Public Health. 2021;198:174–176. doi: 10.1016/j.puhe.2021.07.022 34461451 PMC8318685

[80] Cenin DR, Naber SK, de Weerdt AC, et al. Cost-effectiveness of personalized screening for colorectal cancer based on polygenic risk and family history. Cancer Epidemiol Biomarkers Prev. 2020;29(1):10–21. doi: 10.1158/1055-9965.EPI-18-1123 31748260 PMC7159991

[81] Dixon P, Keeney E, Taylor JC, et al. Can polygenic risk scores contribute to cost-effective cancer screening? A systematic review. Genet Med. 2022;24(8):1604–1617. • This is a systematic review of the current available evidence on the cost effectiveness of combining polygenic risk scores within cancer screening. Ten studies are included: three in colorectal cancer highlighting the current gap in evidence to recommend polygenic risk informed bowel screening. doi: 10.1016/j.gim.2022.04.020 35575786 PMC7614235

[82] Moorthie S, Taylor L, Dennison R, Usher-Smith J. A systems based qualitative analysis exploring the potential to implement risk stratified bowel cancer screening in England. BMC Health Services Research. 2025 Feb 11;25(1):226.. 10.1186/s12913-025-12381-w PMC11812230 39930493

[83] National Screening Committee UDH. UK-NSC explores potential developments in bowel cancer screening; 2024 ­[accessed 2024 May 24]. Available from: https://nationalscreening.blog.gov.uk/2024/02/05/uk-nsc-explores-potential-developments-in-bowel-cancer-screening/

